# The validity, reliability and normative scores of the parent, teacher and self report versions of the Strengths and Difficulties Questionnaire in China

**DOI:** 10.1186/1753-2000-2-8

**Published:** 2008-04-29

**Authors:** Yasong Du, Jianhua Kou, David Coghill

**Affiliations:** 1 Shanghai Mental Health Centre, Shanghai, 200030, ProC; 2 Section of Psychiatry and Behavioural Sciences, Division of Pathology and Neuroscience, University of Dundee, Ninewells Medical School, Dundee, DD1 9SY, UK

## Abstract

**Background:**

The Strengths and Difficulties Questionnaire (SDQ) has become one of the most widely used measurement tools in child and adolescent mental health work across the globe. The SDQ was originally developed and validated within the UK and whilst its reliability and validity have been replicated in several countries important cross cultural issues have been raised. We describe normative data, reliability and validity of the Chinese translation of the SDQ (parent, teacher and self report versions) in a large group of children from Shanghai.

**Methods:**

The SDQ was administered to the parents and teachers of students from 12 of Shanghai's 19 districts, aged between 3 and 17 years old, and to those young people aged between 11 and 17 years. Retest data was collected from parents and teachers for 45 students six weeks later. Data was analysed to describe normative scores, bandings and cut-offs for normal, borderline and abnormal scores. Reliability was assessed from analyses of internal consistency, inter-rater agreement, and temporal stability. Structural validity, convergent and discriminant validity were assessed.

**Results:**

Full parent and teacher data was available for 1965 subjects and self report data for 690 subjects. Normative data for this Chinese urban population with bandings and cut-offs for borderline and abnormal scores are described. Principle components analysis indicates partial agreement with the original five factored subscale structure however this appears to hold more strongly for the Prosocial Behaviour, Hyperactivity – Inattention and Emotional Symptoms subscales than for Conduct Problems and Peer Problems. Internal consistency as measured by Cronbach's α coefficient were generally low ranging between 0.30 and 0.83 with only parent and teacher Hyperactivity – Inattention and teacher Prosocial Behaviour subscales having α > 0.7. Inter-rater correlations were similar to those reported previously (range 0.23 – 0.49) whilst test retest reliability was generally lower than would be expected (range 0.40 – 0.79). Convergent and discriminant validity are supported.

**Conclusion:**

We report mixed findings with respect the psychometric properties of the Chinese translation of the SDQ. Reliability is a particular concern particularly for Peer Problems and self ratings by adolescents. There is good support for convergent validity but only partial support for structural validity. It may be possible to resolve some of these issues by carefully examining the wording and meaning of some of the current questions.

## Background

Mental health problems in children and adolescents result in significant burden and impact not only on the individual child but also their families, schools and communities [1-3]. In China, as in the rest of the world, increasing numbers of children and adolescents are being identified as suffering from a wide range of mental health problems [4-6]. In recent years, China has had a more open policy, and Chinese society has been changing rapidly. There has been a shift from traditional cultural models towards a multi-culture model with traditional ideas increasingly being influenced by different cultures and in particular those from the West [4]. There however remain many differences between contemporary Chinese and Western societies. It seems likely that these differences and the inevitable tensions, between Western and traditional Chinese values, will impact on the lives of children. For the children born during the "one family one child" era life has become very competitive. These are thought by many to have increased the stresses placed upon on the child and to have, potentially, increased the incidence of child and adolescent mental health problems [5]. Also, particularly in South China, where the economy has developed more rapidly, an increasing number of students have been living away from their parents either boarding in schools or living in their teachers' homes. As a consequence teachers have become much more aware of their students emotional functioning and their strengths and difficulties. As a consequence the development and validation of tools that allow teachers views to be considered has become increasingly important [7].

Despite a trend towards increased recognition of children and adolescents with mental health problems, studies of service use generally suggest that only a minority of those with mental health needs are in contact with specialist services [8,9]. Unfortunately strategies for both primary prevention (the prevention of the onset of a condition), and secondary prevention (the identification and treatment of asymptomatic individuals who have already developed risk factors or preclinical disease but in whom the condition is not clinically apparent), are not well developed in child and adolescent mental health fields. It is therefore clearly important that clinicians develop effective, reliable and valid and usable tools that can facilitate the early identification of child and adolescent mental health problems as well as the detection of hidden comorbidities in those presenting with either general physical or mental health problems. Parent, teacher and self report questionnaires can potentially play an important role in this process. A range of questionnaires are available to evaluate behavioural and emotional problems of children and adolescents, several of these have been validated for use in Chinese populations, including the Child Behaviour Checklist, the Rutter Questionnaires, and the Conner's Questionnaires [10-13]. Although these instruments are useful they have several shortcomings. They are felt by many clinicians to be too long, cumbersome to score and to place too great an emphasis on certain behaviours. Their focus on problem behaviours, such as hyperactivity, has also resulted in a reduced acceptance by non-medical professionals. Goodman initially developed the Strengths and Difficulties Questionnaire (SDQ) in the UK [14], it has now been translated into 66 different languages and has become an internationally recognized tool which is extensively used in both research and clinical settings. Use of the SDQ as an assessment of children's behaviour and emotional problems has been supported by the Chairman of the World Psychiatric Association Children's Mental Health Projects. The SDQ has several advantages over the other scales mentioned above. It is relatively short, with only 25 questions and a simple scoring system, making it quick and easy to complete and to score. It has a simple factor structure with good face validity. Perhaps the most important feature of the SDQ is its emphasis on an individual's strengths as well as their difficulties which has resulted in a very broad acceptance by non health professionals, children and their parents.

The structure, normative scoring and psychometric properties of the SDQ have been extensively investigated in samples from the UK and Europe [15-24], the Americas [25-29], Australia [30,31], the Middle East [32-35] and Asia [35,36] Despite these studies having generally supported reliability and validity, several important cross cultural issues have been raised. For example several recent studies have questioned whether the original subscale structure of the SDQ is equally valid in all cultures [21,27,33]. It is therefore essential that the reliability and validity of the SDQ continues to be assessed across differing cultural settings, particularly in situations such as in China, where issues of tradition or social structure and organization may result in subtle alterations in the meaning of specific items which could impact on reliability and validity.

There are currently no published data on the use of the SDQ in China. In order to assist with the preparation and implementation of the World Psychiatric Association Children's Mental Health Projects in Shanghai, a densely populated and rapidly developing urban area, we collected normative data from a large representative community sample in order to address five broad research questions.

• Do the Chinese translations of the parent, teacher and self report versions of the SDQ have the same five subscale factor structure in this population as was demonstrated for the original English version in a UK population?

• What are the mean scores and subscale scores for each version of the questionnaire in this population?

• What are the appropriate normal, borderline and abnormal bandings and cut-off scores for these scales in this population?

• Do the Chinese translations of the SDQ have acceptable reliability in this population?

• Do the Chinese translations of the SDQ have acceptable validity in this population?

## Methods

This is a cross sectional epidemiological study investigating the structure, reliability and validity of the parent, teacher and self report versions on the SDQ.

### Subjects

As it was not possible, for logistic reasons, to include children from across the whole of Shanghai we used a mixture of stratified cluster, random sampling and stratification, to identify children from nursery, primary and secondary schools from 12 of Shanghai's 19 administrative districts. These twelve districts were chosen to be representative of the whole of Shanghai. Within each district schools were randomly chosen and all children within a chosen school were approached. Prior to commencing data collection, we met with all school principals and psychological counselling teachers to explain the significance of the investigation and discuss the research strategy. They in turn informed the students and their parents about the study. We sampled a total of 2128 students aged between 3 – 17 years, including 535 nursery school students, 693 primary school students and 900 secondary school students.

### Research tools

The official Chinese translations of the parent, teacher and self report versions of the Strengths and Difficulties Questionnaire [14] were used. These versions were translated and back-translated by academic staff at the Centre for Clinical Trials and Epidemiological Research at the Chinese University of Hong Kong, and by Iris Tan Mink. Each of these questionnaires includes 25 items, each of which is scored on a three point scale (0 = not true, 1 = somewhat true, 2 = certainly true). Fifteen of the questions ask about difficulties and ten ask about strengths. The ten questions asking about strengths are positively worded. Five of these make up the prosocial behaviours subscale for which, unlike the other four subscales a higher score signifies less problems. The other five positively worded questions are reverse scored. Five subscale scores are generated each of which relates to 5 of the questions. These are; emotional symptoms, conduct problems, hyperactivity/inattention, peer relationship problems and prosocial behaviour. A total difficulties score is calculated by summing four of the subscale scores (emotional symptoms, conduct problems, hyperactivity/inattention and peer relationship problems). In addition, but not used in this study, an impact rating can be generated using separate questions from an impact supplement. In general a high score represents greater difficulties, except for the prosocial scale score where a lower score indicates greater difficulties. General information on the SDQ, the Chinese versions, and the SDQ scoring can be found online[37,38]. Parents and teachers were asked to rate the behavioural and emotional aspects of the child's behaviour over the past six months as per their general observations of the child, young people aged 11 – 17 were asked to rate themselves over the past six months. Parents were also asked to complete the Chinese version of the Conner's Parent Symptoms Questionnaire (PSQ) [39].

### Data Collection

Parents, teachers who knew the children well and young people aged between 11 and 17 years, completed questionnaires. Questionnaires were completed in the classrooms at the children's schools, guided by a trained psychological counselling teacher. If whilst completing the questionnaire either the parent the teacher or the young person had doubts about how to proceed the psychological counselling teachers would explain. Each parent and teacher completed the questionnaire alone, and handed in the questionnaires to the psychological counselling teachers. We received a total of 2,101 (98.7%) questionnaires for parents, 2,123 (99.7) from teachers and 816 (90.6%) from young people. A questionnaire was considered invalid if answers were missing for one or more questions. Only subjects with complete parent and teacher data were analysed and data from the one subject younger than 3 years and the one subject older than 17 years were excluded. One thousand nine hundred and sixty five subjects had complete parent questionnaires and teacher questionnaires (93.5% of the parent questionnaires and 92.5% of the teacher questionnaires) and 690 subjects had complete self report, parent and teacher questionnaires (84.6% of eligible subjects). There were no differences with respect district, age or gender between those with complete and incomplete questionnaires (social class data were not available) and the sample was representative of the Shanghai population with respect age and gender distribution. There were no other exclusion criteria. Retest data was collected from parents and teachers for 45 students six weeks later (practical limitations precluded a shorter re-testing interval).

### Statistical analysis

We established the database of the raw data in FoxPro; data description and statistical analyses were performed by SPSS (versions 11.0 and 14.0). Statistical analyses were conducted on unweighted data. Normative data is presented descriptively. Distributions of raw scores were used to determine the cut-off scores to identify normal, borderline and abnormal bandings. Where appropriate analyses were repeated for two age bandings (3 – 10 years and 11 – 17 years). A principle components analyses was conducted to investigate the subscale structure of the scales. Reliability was assessed from analyses of internal consistency using Cronbach'sα, inter-rater agreement, and temporal stability (test retest reliability) for which test-retest reliability ≥ 0.7 is deemed to be satisfactory [40]. Structural validity was assessed via cross scale correlations. Convergent validity was assessed by calculating correlations between the parent completed SDQ and the parent completed PSQ, Discriminant validity was assessed by comparing 47 subjects from the normative sample with 47 age and gender matched ADHD outpatients using receiver operating characteristic (ROC) curves employing area under the curve (AUC) as an index of discriminant ability. For the AUC a score ≤ 0.6 suggests that discrimination is no better than chance; 0.6 – 0.75 is fair; 0.75 – 0.90 is good, 0.90 – 0.97 is very good and 0.97 – 1. 0 is excellent [41].

## Results

Complete parent and teacher data were available for 1965 children and complete parent, teacher and self report data were available for 690 cases. There were no differences with respect to age and gender between those cases with and without complete data. Data on social class were not available. These data were used to generate the following results.

### Scale means, age and gender effects

The mean SDQ subscale scores for parent, teacher and self ratings subdivided by age-band (3 to 10 years and 11 to 17 years) and gender are presented in tables 1, 2 and 3 respectively. For all three raters boys of all ages were rated as having statistically significantly greater difficulties on the total problems score and on the conduct problems, hyperactivity/inattention, peer problems, and prosocial behaviour subscales with one exception; parent ratings of peer problems in the younger age group showed no gender differences. On the emotional symptoms subscale younger but not older girls were rated as having statistically significantly greater difficulties on the parent rated scale. There were no gender differences seen on this subscale on the teacher or self reported self reported scales (all significant p values ≤ 0.001).

**Table 1:** Mean Subscale Scores by age and gender for the parent completed SDQ in a community sample of 3 – 17 year old Chinese children

	Mean Scores (Std. Dev.)								
SDQ Scale Parent	Total (n = 1965) [Male n = 950, Female = 1015]			3 – 10 years (n = 1217) [Male n = 595, Female = 622]			11 – 17 years (n = 748) [Male n = 355, Female = 393]		

Emotional Symptoms	1.97 (1.83) [UK 1.9]*	Male	1.84 (1.77)	2.09 (1.83)	Male	1.94 (1.75)	1.76 (1.83)	Male	1.66 (1.81)
		Female	2.09 (1.88)		Female	2.25 (1.89)		Female	1.85(1.85)
Conduct Problems	1.57 (1.45) [UK 1.6]*	Male	1.77 (1.55)	1.59 (1.42)	Male	1.80 (1.52)	1.53 (1.50)	Male	1.72 (1.60)
		Female	1.39 (1.33)		Female	1.40 (1.30)		Female	1.35(1.40)
Hyperactivity – Inattention	4.22 (2.42) [UK 3.5]*	Male	4.64 (2.44)	4.49 (2.45)	Male	4.88 (2.47)	3.77 (2.30)	Male	4.25 (2.35)
		Female	3.83 (2.33)		Female	4.13 (2.38)		Female	3.33 (2.16)
Peer Problems	2.71 (1.67) [UK 1.5]*	Male	2.84 (1.69)	2.71 (1.70)	Male	2.70 (1.74)	2.72 (1.62)	Male	3.05 (1.59)
		Female	2.59 (1.64)		Female	2.71 (1.67)		Female	2.42 (1.58)
Prosocial Behaviour	7.14 (1.98) [UK 8.6]*	Male	6.80 (2.01)	7.16 (1.91)	Male	6.84 (1.94)	7.13 (2.07)	Male	6.76 (2.12)
		Female	7.46 (1.89)		Female	7.47 (1.83)		Female	7.46 (1.97)
Total Difficulties	10.48 (4.93) [UK 8.4]*	Male	11.09 (4.99)	10.89 (4.84)	Male	11.32(4.89)	9.77 (5.00)	Male	11.03 (5.17)
		Female	9.90 (4.80)		Female	10.49 (4.77)		Female	8.27 (5.11)

* UK norms as reported in [38]

**Table 2:** Mean Subscale Scores by age and gender for the teacher completed SDQ in a community sample of 3 – 17 year old Chinese children

	Mean Scores (Std. Dev.)								
SDQ Scale Teacher	Total (n = 1965) [Male n = 950, Female = 1015]			3 – 10 years (n = 1217) [Male n = 595, Female = 622]			11 – 17 years (n = 748) [Male n = 355, Female = 393]		

Emotional Symptoms	1.78 (1.79) [UK 1.4]*	Male	1.75 (1.78)	1.76 (1.82)	Male	1.75 (1.80)	1.81 (1.75)	Male	1.75 (1.76)
		Female	1.81 (1.80)		Female	1.77 (1.84)		Female	1.85 (1.85)
Conduct Problems	1.38 (1.63) [UK 0.9]*	Male	1.73 (1.82)	1.32 (1.60)	Male	1.67 (1.78)	1.47 (1.68)	Male	1.72 (1.60)
		Female	1.06 (1.36)		Female	0.99 (1.31)		Female	1.16 (1.44)
Hyperactivity – Inattention	3.84 (2.73) [UK 2.9]*	Male	4.65 (2.80)	3.96 (2.71)	Male	4.70 (2.80)	3.63 (2.72)	Male	4.52 (2.80)
		Female	3.08 (2.42)		Female	3.23 (2.44)		Female	2.83(2.87)
Peer Problems	2.40 (1.76) [UK 1.4]*	Male	2.66 (1.84)	2.22 (1.73)	Male	2.47 (1.83)	2.67 (1.78)	Male	2.94 (1.83)
		Female	2.15 (1.65)		Female	1.98 (1.60)		Female	2.42 (1.69)
Prosocial Behaviour	6.86 (2.47) [UK 7.2]*	Male	6.29 (2.55)	6.80 (2.42)	Male	6.33 (2.49)	6.99 (2.55)	Male	6.24 (2.66)
		Female	7.40 (2.27)		Female	7.25 (2.26)		Female	7.67 (2.24)
Total Difficulties	9.40 (5.67) [UK 6.6]*	Male	10.78 (6.03)	9.26 (5.60)	Male	10.60 (5.98)	9.58 (5.77)	Male	11.03 (6.11)
		Female	8.10 (5.04)		Female	7.97 (4.90)		Female	8.27 (5.11)

* UK norms as reported in [38]

**Table 3:** Mean Subscale Scores by gender for the self completed SDQ in a community sample of 11–17 year old Chinese children

SDQ Scale Self	Mean Scores (Std. Dev.)		
	
	Total (n = 690) [Male n = 326, Female = 364]		
Emotional Symptoms	2.30 (1.96) [UK 2.8]*	Male	2.36 (1.97)
		Female	2.25 (1.94)
Conduct Problems	2.16 (1.44) [UK 2.2]*	Male	2.35 (1.50)
		Female	1.99 (1.36)
Hyperactivity – Inattention	3.32 (2.08) [UK 3.8]*	Male	3.65 (2.09)
		Female	3.03 (2.02)
Peer Problems	2.85 (1.67) [UK 1.5]*	Male	3.22 (1.69)
		Female	2.52 (1.58)
Prosocial Behaviour	7.32 (1.92) [UK 8.0]*	Male	6.80 (1.94)
		Female	7.78 (1.78)
Total Difficulties	10.60 (4.83) [UK 10.3]*	Male	11.54 (4.95)
		Female	9.75 (4.57)

* UK norms as reported in [38]

For parent ratings there was a main effect of age on the emotional symptoms [F (1, 1963) = 11.8, p < .001] and hyperactivity/inattention [F (1, 1963) = 40.7, p < .001] subscales. For both of these subscales the scores decreased as age increased. There was no main effect of age on parent rated conduct problems, peer problems or prosocial behaviour. There were gender × age interactions for peer problems [F(2,1962) = 11.7, p < .001] whereby the boys peer relations were rated as getting worse as they got older and girls were rated as improving.

For teacher ratings there was a main effect of age on hyperactivity/inattention [F (1, 1963) = 12.7, p < .001], peer problems [F (1, 1963) = 34.8, p < .001] and prosocial behaviour [F (1, 1963) = 14.2, p < .001]. Hyperactivity/inattention and prosocial behaviour were adjudged to have improved as the children got older, peer relations were rated as worse for older children than for younger children. There was no main effect of age on teacher rated emotional symptoms or conduct problems. There was gender × age interaction for teacher rated prosocial behaviour [F (2, 1962) = 12.7, p < .01] and of the teacher reported subscales whereby boys older boys were rated as less prosocial and older girls as more prosocial.

Age effects were not calculated for the self reports due to the constricted age range in this sample.

### Bandings and cut-offs

Bandings and cut-offs were estimated from the distributions of raw values in the manner described by Woerner, et al [15]. For the total difficulties scores cut-offs were calculated with the intention of placing approximately 10% of the sample with the most extreme scores in the "abnormal" banding, the next 10% in the "borderline" banding and the remaining 80% in the "normal" banding. As prevalence's for individual disorders are necessarily lower than those for any disorder it was felt more appropriate to place a slightly lower percentage of subjects in the abnormal and borderline bandings for each of the subscales therefore cut-offs were determined for each such that approximately 85% of subjects were placed in the normal banding and 7.5% in each of the abnormal and borderline bandings. However since each of the subscales can only have a limited number of scores (i.e. 11, between 0 and 10) the actual percentages could only be approximated. These bandings are shown in table 4 along with the actual percentage of subjects in each of the three banding categories. In view of the extended age range of the sample these bandings were also calculated separately for younger and older age ranges for the parent and teacher completed scales. The bandings for the different age groups were very similar with few differences (data not shown).

**Table 4:** Recommended bandings of raw scores obtained from a sample of 3 – 17 year old Chinese children

Informant	Scale	Normal range		Borderline range		Abnormal range	
		
		Raw score	Exact %	Raw score	Exact %	Raw score	Exact %
Parent	Total Difficulties	0 – 14	79.0	15 – 16	8.9	17 – 40	12.1
	Emotional Symptoms	0 – 3	81.1	4	8.9	5 – 10	10.0
	Conduct Problems	0 – 2	79.0	3	11.9	4 – 10	9.1
	Hyperactivity Inattention	0 – 6	82.3	7	6.4	8 – 10	11.3
	Peer problems	0 – 4	85.9	5	8.8	6 – 10	5.3
	Prosocial behaviours	10 – 6	78.7	5	12.2	4 – 0	9.1
Teacher	Total Difficulties	0 – 13	78.5	14 – 17	11.7	18 – 40	9.8
	Emotional Symptoms	0 – 3	83.6	4	8.5	5 – 10	7.9
	Conduct Problems	0 – 2	80.0	3	9.5	4 – 10	10.5
	Hyperactivity Inattention	0 – 6	83.1	7 – 8	9.0	9 – 10	7.9
	Peer problems	0 – 4	88.0	5	6.7	6 – 10	5.3
	Prosocial behaviours	10 – 5	83.8	4	7.0	3 – 0	9.2
Self	Total Difficulties	0 – 14	79.0	15 – 17	11.0	18 – 40	10.0
	Emotional Symptoms	0 – 4	84.6	5	8.3	6 – 10	7.1
	Conduct Problems	0 – 3	81.5	4	11.1	5 – 10	7.4
	Hyperactivity Inattention	0 – 5	84.5	6	8.1	7 – 10	7.4
	Peer problems	0 – 4	83.6	5	10.4	6 – 10	6.0
	Prosocial behaviours	10 – 6	81.7	5	10.5	4 – 0	7.8

### Reliability

#### Internal consistency

The Cronbach's α coefficients for the parent and teacher SDQ subscales and total score are reported in table 5. As above data from Goodman et al. (2001) have been included in this table for comparison. Overall the α coefficients were lower than hoped for. The α coefficient directly reflects the degree of the internal consistency of the factors and an α ≥ 0.70, is generally considered to indicate good internal consistency sufficient for group comparison [42]. For the parent subscales only the hyperactivity/inattention (α = 0.76) subscale had an α ≥ 0.70 with the other α coefficients ranging between 0.30 and 0.68. The alphas for the teacher subscales were constantly higher than those for the parent subscales however good reliability was only found for the hyperactivity/inattention (α = 0.82) and prosocial behaviours (α = 0.83) subscales. The other subscales alphas ranged between 0.48 and 0.63. For the self reported scale the subscale α coefficients were lower than for the other two informants and none of the subscales had an α coefficient > 0.7 (range 0.30 – 0.64).

**Table 5:** Reliability coefficients for Parent, Teacher and Self rated SDQ in a community sample of 3 – 17 year old Chinese children

	Reliability Correlations – Cronbach's α		
SDQ Scale	Parent (N = 1965)	Teacher (N = 1965)	Self (N = 690)

Total Difficulties Score	0.59	0.60	0.57
Emotional Symptoms	0.60 (0.67)*	0.63 (0.78)	0.59 (0.66)
Conduct Problems	0.48 (0.63)	0.63 (0.74)	0.33 (0.60)
Hyperactivity – Inattention	0.76 (0.77)	0.82 (0.88)	0.64 (0.67)
Peer Problems	0.30 (0.57)	0.48 (0.70)	0.30 (0.41)
Prosocial Behaviour	0.68 (0.65)	0.83 (0.84)	0.66 (0.66)

*Comparative data from Goodman, 2001 in brackets for comparison

These analyses were repeated for the two main age bands (3 – 10 years and 11 to 17 years). The results of these analyses were very similar to those for the whole group and are not reported further (range for 3 – 10 years, parent 0.29 – 0.74, teacher 0.45 – 0. 84, self 0.29 – 0.62, range for 11 to 17 years, parent 0.32 – 0.77, teacher 0.49 – 0. 83, self 0.30 – 0.65).

#### Inter-rater correlations

The inter-rater correlations between parents and teachers are reported in table 6. To keep consistency with the Goodman [16] paper the mean cross-informant correlations for other similar measures based on the meta-analysis conducted by Achenbach et al. [43] have been included for comparison. These data were also analyzed by age. The correlations were between parents and teachers were consistently higher for the younger children (3 – 10 years) than for the older children (11 – 17 years) (data not shown).

**Table 6:** Inter-rater correlations for SDQ scores in a community sample of 3 – 17 year old Chinese children

SDQ Scale	Pearson (spearman) Inter-rater Correlations		
	
	Parent X Teacher (n = 1965)	Parent X Self (n = 1965)	Teacher X Self (n = 690)
Emotional Symptoms	0.23 (0.25)	**0.42 **(0.41)	**0.29 **(0.31)
Conduct Problems	**0.31 **(0.32)	**0.39 **(0.38)	**0.34 **(0.32)
Hyperactivity – Inattention	**0.44 **(0.44)	**0.37 **(0.39)	**0.38 **(0.38)
Peer Problems	**0.29 **(0.30)	**0.36 **(0.33)	**0.35 **(0.37)
Prosocial Behaviour	**0.27 **(0.27)	**0.40 **(0.39)	**0.31 **(0.28)
Total Difficulties	**0.36 **(0.46)	**0.49 **(0.48)	**0.42 **(0.33)
Pearson meta-analytic mean for other measures reported by Achenbach et al. (1987)	0.27	0.25	0.20

Note: All SDQ correlations significant at *p *< 0.001. Correlations in bold type are ≥ to the meta-analytic mean reported by Achenbach et al. (1987).

#### Test-retest reliability

Parents and teachers of sixth grade students completed the SDQ for a second time 6 weeks after their first completion. Test retest correlations of ≥ 0.7 are generally considered reliable. The correlations between these scores are reported in table 7. All the coefficients were statistically significant (P < 0.001).

**Table 7:** Test Retest Reliability of Parent and Teacher SDQ in a community sample of 3 – 17 year old Chinese children

SDQ Scale	Parent (N = 45)	Teacher (N = 45)
Emotional Symptoms	0.47	0.40
Conduct Problems	0.70	0.50
Hyperactivity – Inattention	0.48	0.64.
Peer Problems	0.79	0.58
Prosocial Behaviour	0.43	0.50
Total Difficulties Score	0.72	0.55

Note all correlations significant p < 0.001

### Validity

#### Principle Components Analyses

The results of the rotated principal components analyses with subsequent Varimax rotation for the parent, teacher and self rated SDQs are detailed in tables 8, 9 and 10 respectively. In each analysis a fixed 5 component solution was chosen in order to obtain comparability with the original SDQ papers.

**Table 8:** Principle Components analysis of parent rated SDQ scores in a community sample of 3 – 17 year old Chinese children (N = 1965)

**Total Variance Explained**	**Component 1 "Prosocial" 17.2%**	**Component 2 "Hyper/Innatt" 9.4%**	**Component 3 "Emotional" 7.5%**	**Component 4 "Conduct 1" 5.6%**	**Component 5 "Conduct 2" 4.6%**
**(Question number) Question**					

**(1) Considerate**	.524	-.306			
**(4)Shares**	.565				
**(9)Helpful**	.681				
**(17)Kind to kids**	.632				
**(20)Helps out**	.655				
**(2)Restless**		.486		.571	
**(10)Fidgeting**		.501		.522	
**(15)Distractible**		.722			
**(21)Reflective**		.684			
**(25)Persistent**		.729			
**(3)Somatic**			.334		.359
**(8)Worries**			.567		
**(13)Unhappy**			.520	.462	
**(16)Clingy**			.684		
**(24)Fears**			.686		
**(5)Tempers**				.736	
**(7)Obedient**				.428	
**(12)Fights**	**-.392**			.479	.368
**(18)Argues with adults**					.678
**(22)Spiteful**					.514
**(6)Solitary**			.584		
**(11)Good friend**	**-.375**				
**(14)Popular**	**-.577**				
**(19)Bullied**					.602
**(23)Best with adults**					

Rotation Method: Varimax with Kaiser Normalization.

Rotation converged in 7 iterations.

**Table 9:** Principle Components analysis of teacher rated SDQ scores in a community sample of 3 – 17 year old Chinese children (N = 1965)

**Total Variance Explained**	**Component 1 "Prosocial" 25.6%**	**Component 2 "Hyper/Innatt/Conduct" 9.0%**	**Component 3 "Hyper/Innatt" 8.4%**	**Component 4 "Emotional" 5.1%**	**Component 5 "Peer" 4.4%**
**(Question number) Question**					

**(1) Considerate**	-.676		-.351		
**(4)Shares**	-.722				
**(9)Helpful**	-.779				
**(17)Kind to kids**	-.703				
**(20)Helps out**	-.710				
**(2)Restless**		.645	.498		
**(10)Fidgeting**		.660	.437		
**(15)Distractible**		.390	.677		
**(21)Reflective**	.488		.643		
**(25)Persistent**	.437		.615		
**(3)Somatic**		.473		.322	
**(8)Worries**				.615	
**(13)Unhappy**		.470		.511	
**(16)Clingy**				.689	
**(24)Fears**				.717	
**(5)Tempers**		.695			
**(7)Obedient**	.510	.423			
**(12)Fights**		.702			
**(18)Argues with adults**		.354			.474
**(22)Spiteful**					.462
**(6)Solitary**				.535	.333
**(11)Good friend**	.494				.366
**(14)Popular**	.654				
**(19)Bullied**				.314	.513
**(23)Best with adults**					.496

Rotation Method: Varimax with Kaiser Normalization.

Rotation converged in 10 iterations.

**Table 10:** Principle Components analysis of self rated SDQ scores in a community sample of 11 – 17 year old Chinese children (N = 690)

**Total Variance Explained**	**Component 1 "Prosocial" **15.6%	**Component 2 "Emotional" **9.0%	**Component 3 "Hyper/Innatt" **6.2%	**Component 4 "Mixed1" **5.2%	**Component 5 "Mixed2" **4.8%
**(Question number) Question**					

**(1) Considerate**	.617				
**(4)Shares**	.537				
**(9)Helpful**	.693				
**(17)Kind to kids**	.507				-.355
**(20)Helps out**	.622				
**(2)Restless**			.596		
**(10)Fidgeting**		.431	.433		
**(15)Distractible**			.709		
**(21)Reflective**			.510		
**(25)Persistent**	-.398		.552		
**(3)Somatic**		.446			
**(8)Worries**		.675			
**(13)Unhappy**		.724			
**(16)Clingy**		.365	.301		-.612
**(24)Fears**		.541		.329	
**(5)Tempers**		.530			.413
**(7)Obedient**					
**(12)Fights**					.521
**(18)Argues with adults**			.320	.466	
**(22)Spiteful**				.570	
**(6)Solitary**	-.302	.398			
**(11)Good friend**					
**(14)Popular**	-.583				
**(19)Bullied**				.596	
**(23)Best with adults**				.507	

Rotation Method: Varimax with Kaiser Normalization.

Rotation converged in 9 iterations.

For the parent ratings the prosocial behaviour, hyperactivity/inattention and emotional symptoms items loaded on the predicted components, the conduct items loaded onto two separate components. Two of the peer problems items (good friend and popular) loaded onto the prosocial component, "good friend" loaded onto the emotional symptoms component and "bullied" loaded onto one of the conduct components. The "Best with adults" question did not load onto any of the components. The other three peer problems items (solitary, popular, bullied) each loaded independently onto one of the other components. Three items (somatic, restless and fidgeting) also loaded onto conduct components with higher loadings than they did onto their predicted component.

For the teacher ratings the outcome was less clear. The five prosocial items loaded onto a single component on which there were also high loadings for five other positively worded questions two hyperactivity/inattention items (reflective, persistent) one conduct item (obedient) and two peer problems items (good friend and popular). All 5 hyperactivity/inattention items loaded onto a single component however two items had higher loadings on another component that also included the highest loadings for two conduct symptoms (tempers and fights) 1 emotional symptom item (somatic) and moderate loading for another two conduct items (obedient and argues with adults) that however loaded higher onto other scales. The four other emotional symptoms items had their highest loading onto a single component. Four of the peer problems items (bullied, best with adults, good friend and popular) loaded onto a single component along with two conduct items (argues with adults and spiteful) however two of the peer problems items (good friend and popular) loaded more highly onto the prosocial behaviours component. Both the parent and teacher rated "prosocial" components could also have been labelled as a "positive" component as the additional items which loaded highly on them were all positively worded.

For the self reported ratings prosocial behaviour, hyperactivity/inattention and emotional symptoms items again loaded on the predicted components. There were two less well defined "mixed" components the first of which included two conduct items (Argues with adults and spiteful), one emotional symptoms item (fears) and two peer relationships items (bullied and best with adults), a second "mixed" component included two conduct items (tempers and fights) and to items negatively correlated with these one from the emotional subscale (clingy) and a prosocial item (kind to kids).

#### Age effects

The parent and teacher principle components analyses were repeated with the sample split into two age groupings (3 – 10 years and 11 to 17 years). The results from each of these analyses were very similar to those described above (data not shown) and are not discussed further.

#### Cross-Scale Correlations

The cross-scale correlations between the three psychopathological subscales are reported separately for each informant in table 11. As a comparison the figures for the same analysis from the original UK description of the psychometric properties of the SDQ [16] have been included. As expected the conduct – hyperactivity/inattention correlations (parent = .46, teacher = .61, self = .39) are considerably higher than either the conduct – emotional (parent = .22, teacher = .22, self = .27) or the hyperactivity/inattention – emotional ones (parent = .21, teacher = .19, self = .33).

**Table 11:** Cross scale correlations for Parent, Teacher and Self Completed SDQs in a community sample of 3 – 17 year old Chinese children

		Pearson Cross-Scale Correlations		
Informant	N	Emotion – Conduct	Emotion- Hyper/Inatt	Conduct – Hyper/Inatt

Parent	1965	0.22 (0.30)*	0.21 (0.26)	0.46 (0.50)
Teacher	1965	0.22 (0.21)	0.19 (0.24)	0.61 (0.61)
Self	690	0.27 (0.33)	0.33 (0.31)	0.39 (0.53)

*Comparative figures from Goodman, 2001 are in brackets for comparison

#### Convergent validity

The Conner's Parent Symptom Questionnaire (PSQ) is frequently used to evaluate children's behaviour [10]. Su has developed and validated a Chinese version of the PSQ [39]. We conducted convergent validity analysis between SDQ and PSQ. All the parents were asked to complete the PSQ at the same time as completing the SDQ. Data was available for 1940 subjects. The scores of the SDQ and PSQ subscales were correlated with each other. The results of this analysis are reported in Table 12. As expected the correlations are highest for matching subscales and between externalizing – externalizing pairs and internalizing – internalizing pairs, lower for externalizing – internalizing pairs and in-between for the peer and prosocial subscales of the SDQ and subscales of the PSQ which does not attempt to measure these domains. Similarly the correlations between the physical and mental problems subscale of the PSQ and the SDQ subscales are low.

**Table 12:** Correlations between parent rated SDQ and parent rated PSQ in a community sample of 3 – 17 year old Chinese children

N = 1940	PSQ Scale						
SDQ Scale	Conduct problems	Learning problems	Physical and mental problems	Impulsivity- hyperactivity	Anxiety	Hyperactivity index score	Total score

Emotional Symptoms	0.26	0.29	0.26	0.18	0.46	0.28	0.36
Conduct Problems	0.53	0.31	0.11	0.42	0.06	0.44	0.44
Hyperactivity – Inattention	0.41	0.58	0.11	0.56	0.08	0.61	0.56
Peer Problems	0.15	0.21	0.10	0.11	0.18	0.16	0.19
Prosocial Behaviour	-0.27	-0.51	0.22	-0.14	-0.12	-0.21	-0.25
Total Difficulties Score	0.51	0.54	0.22	0.53	0.30	0.60	0.63

Note all correlations significant p < 0.001

#### Discriminant validity

We compared 48 respondents from the normative sample with 47 ADHD outpatients matched for age and gender. As expected the hyperactivity/inattention subscale and total difficulties scores were scored higher by all raters for the ADHD group, than for the control group. Parents and teachers also scored the ADHD group higher for conduct problems and the teachers scored them higher for emotional symptoms. ROC analyses supported the ability of the Chinese SDQ to discriminate between these two groups. For this purpose the underlying assumption was that children with ADHD were substantially more likely to have problems with hyperactivity/inattention, conduct, peer relationships, prosocial behaviours and total difficulties than the control children. In ROC analyses sensitivity and specificity are calculated for all possible cut-offs on the questionnaire. These are then combined to give a statistic the "area under the curve" (AUC). Values for AUC are between 0 and 1.0. The convention for interpreting AUC is that an AUC ≤ 0.6 suggests that discrimination is no better than chance; 0.6 – 0.75 is fair; 0.75 – 0.90 is good, 0.90 – 0.97 is very good and 0.97 – 1. 0 is excellent [41]. The results for the ROC analyses are summarized in table 13. All of the SDQ scales and subscales, except for the parent scored peer relations and prosocial behaviours subscales, discriminated between the ADHD and control cases better than chance. Whilst most of the AUCs were in the "fair" range (0.6 – 0.75) several (parent and teacher Hyperactivity – Inattention, and teacher hyperactivity -inattention, conduct problems and total difficulties), were "good" (0.75 – 0.90). The teacher ratings were significantly better at discriminating hyperactivity – inattention, conduct problems and total difficulties than either the parental or the self report ratings.

**Table 13:** Ability of different Strength and Difficulties Questionnaire Scores to distinguish between community and ADHD samples

Rater	Subscale	Area Under the Curve (AUC)	Asymptotic 95% confidence intervals	
			
			Lower Bound	Upper Bound
Parent	Hyperactivity – Inattention	0.77	0.71	0.83
	Conduct Problems	0.68	0.61	0.75
	Peer Problems	0.55	0.47	0.63
	Prosocial Behaviours	0.39*	0.29	0.49
	Total Difficulties	0.69	0.62	0.76

Teacher	Hyperactivity – Inattention	0.90	0.86	0.95
	Conduct Problems	0.87	0.82	0.92
	Peer Problems	0.69	0.61	0.77
	Prosocial Behaviours	0.67	0.60	0.74
	Total Difficulties	0.91	0.87	0.95

Self	Hyperactivity – Inattention	0.70	0.61	0.78
	Conduct Problems	0.61	0.52	0.70
	Peer Problems	0.69	0.61	0.77
	Prosocial Behaviours	0.65	0.57	0.74
	Total Difficulties	0.72	0.64	0.80

* a significant AUC score < 0.5 indicates worse than chance

## Discussion

The normative scores, bandings and cut-offs and the psychometric properties of the Chinese version of the SDQ were evaluated for a representative sample of children and adolescents aged between 3 and 17 years from 12 of the 19 districts of Shanghai. The collection and description of normative data within specific populations is important as differing means are possible both as a consequence of actual differences in the prevalence of particular difficulties between different populations and as a result of cultural biases and expectations as to what is "normal" on the part of raters with differing backgrounds and experiences. In general the Chinese normative data closely resembles that from the UK [44]. In particular the age and gender patterns were similar to those seen in the UK sample. It was however noticeable that the Chinese scores for the peer problems subscale were consistently higher than those for the UK. As we failed to replicate the "peer problems" grouping in our principle components analysis it seems likely that these differences in scoring may reflect a difference in meaning for these questions rather than a true difference in peer relationships. In addition the Chinese teachers also tended to rate conduct problems, hyperactivity – inattention, and total difficulties somewhat higher than their UK counterparts. With respect to the bandings and cut-off scores for the total scale and subscales there were again only minor differences. The teachers higher scoring on several subscales was associated with slightly broader "normal" bands meaning that children some Chinese children who would be rated as "normal" would have been within the "borderline" band had they had the same score in a UK sample.

Whilst these normative data provide important information for future researchers and clinicians who wish to use the SDQ in China, the overall usefulness of these scales in this setting is dependent on the SDQ, originally designed for use in a Western cultural setting, proving to be reliable and valid in a Chinese population. Our findings extend and partially replicate previous findings from community and clinic samples from around the world and suggest reasonable but not unequivocal validity and reliability.

When the psychometric properties of the SDQ have previously been examined in differing cultural contexts the results have generally supported reliability and validity. However several important cross cultural issues have been raised [33]. Several studies have supported the original five factor structure of the SDQ in both clinical and epidemiological samples [15,20,30,32,45-47], others have raised questions about the structural validity of this model. Studies across several cultures have reported low internal consistencies for the parent and self report Conduct Problems subscale and the self-report Peer Problems subscale [16,18-21,47-49]. These may simply be due to the fact that each subscale only contains 5 questions or they may suggest that, at least in some cultures, these subscales represent and tap into more heterogeneous constructs than originally intended. Several recent studies have questioned the original subscale structure of the SDQ and more specifically whether it is equally applicable across differing cultures. Thabet et al [33] conducted a confirmatory factor analysis of the Arabic version of the SDQ scored by parents of children within the Gazza Strip. Whilst there was some support for the original 5 factor structure they found that certain items appeared to have a different function or meaning than is seen in western children and their parents. These included; being unhappy, scared, and distractible, stealing, and being picked on or bullied. As a consequence the emotional and peer relationship subscales and the total difficulties scores seemed to be either more heterogeneous or more multifactorial than is typically seen in western cultures. Dickey and Blumberg [27] in a US sample also failed to replicate the original five factor structure. They concluded that a three factor model, consisting of externalizing problems, internalizing problems and positively worded items, was the most stable and best accounted for their parent reported data. Koskelainen et al [48] also reported a three factor solution as the most adequate representation for a Finnish sample. Using the self report version of the Dutch SDQ Muris et al [49] reported a four-factor solution (Emotional Symptoms, Prosocial Behaviour including positively worded items from other scales, Hyperactivity-Inattention and a mixed Peer Problems -Conduct Problems scale) as the most satisfactory solution. Most recently Palmieri and Smith [29] used confirmatory factor analysis to investigate three models of the SDQs factor structure using data from a US sample of custodial grandmothers and found that the best representation of the latent structure was provided by a model which included the original five factors and an additional factor comprising a "positive construal" factor made up from the positively worded questions.

In our Chinese sample the principle components analyses in the main support the Hyperactivity-Inattention, Emotional and Prosocial subscales but provide less support for the Conduct and Peer Problems subscales. There was also some support for a positive construal component as suggested by Palmieri & Smith [29]. It is possible that this pattern of results reflects the underlying nature of the subscales and represent a greater cross cultural acceptance and consistency of what should be regarded as a prosocial behaviour, and as a behaviour indicative of hyperactivity/impulsivity disorders (i.e. ADHD) and emotional disorders (i.e. anxiety and depression), than there is about what types of behaviours indicate the presence of oppositionality and conduct problems and positive peer relationships. The problems with the peer problems subscale were, as would be expected mirrored by low estimates of internal consistency for this subscale across all three raters.

Other aspects of reliability as measured by internal consistency were also rather disappointing. Other than for prosocial behaviours and hyperactivity/inattention all of the internal consistency coefficients for the Chinese sample were all somewhat lower than those reported in the original analysis of the psychometric properties of the SDQ [16]. None of the self reported measures had an α > 0.70 and only the hyperactivity – inattention subscale for the parent scale and the hyperactivity – inattention and the prosocial behaviour scales for the teacher scale reached this level. As was previously reported by Goodman [16] the reliabilities for the teachers were consistently higher than those for the parents and both of these were more reliable than the self report scale. Inter-rater correlations were, however, reasonable and indeed in this respect our sample was again very similar to that Goodman's [16] with all but one of the inter-rater correlations exceeding the meta-analytic mean reported by Achenbach et al. [43].

The validity of the Chinese versions of the SDQ was supported by the cross scale correlations, which were very similar to those previously reported by Goodman in a UK sample [16]. The convergent validity with the Connors Parent Symptom Questionnaire and the discriminant validity as measured by the ability of the Chinese SDQ to discriminate between a community sample of children and children with ADHD were also very good. With respect to discriminant validity the AUC values from this sample are similar to those previously reported for a German sample [45].

The SDQ has generally been thought of as a screening instrument rather than a measure of outcome. We are aware however of several clinical centres using the SDQ as an outcome measure e.g[50]. Unfortunately the test-retest reliability of the SDQ, a prerequisite for measuring outcome, has not yet been extensively investigated. A test-retest reliability ≥ 0.7 is generally reported as satisfactory [40]. Goodman [51] reported data from a small sample of UK parents retested 3–4 weeks after initial testing, the intra-class correlations ranged from 0.44 for the "burden" item from the impact scale to 0.85 for total difficulties. Unfortunately the coefficients for the five subscales are not reported. Hawes & Dadds [30] reported correlations for retesting on the parent instrument after 12 months. As they acknowledge correlations over this period of time will reflect real changes in the child's behaviour due to development, environmental changes etc., as well as instrument instability, and as a consequence they would be expected to under-estimate stability. It is therefore notable that these correlations, which ranged between 0.61 for peer problems and 0.77 for hyperactivity-inattention, were as high as they were. Indeed the test retest reliabilities for the Emotional Symptoms, Hyperactivity – Inattention, Prosocial Behaviour subscales and for the Total Difficulties score for this Australian sample were larger than those reported here. Only Muris et al [47] have reported the test retest stability of the self report scale. They obtained retest data from 91 young people and their parents two months after initial testing. With the exception of the self reported prosocial subscale correlations for both informants on all subjects the intra-class correlations were all above 0.70. As far as we are aware ours is the first study to report the test retest reliability of the teacher SDQ. Our results are less positive than previously reported. Despite the intra-class correlations all being significant with p < 0.001, they were lower than expected ranging between 0.40 for teacher rated Emotional Symptoms to 0.79 for parent rated Peer Problems with only two other correlations ≥ 0.70 (parent rated Conduct Problems and Total Difficulties).

It must be noted that Shanghai is a densely populated and rapidly developing urban area and that these findings may not generalize to other more rural provinces.

## Conclusion

In summary we report mixed findings with respect the psychometric properties of the Chinese translation of the SDQ. The structural analysis suggests that whilst there is support for the Prosocial behaviour, Hyperactivity/Inattention and Emotional Problems subscales there appear to be differences in the way the Chinese interpret the questions relating to Conduct and Peer Problems. These differences may also underpin the lower internal consistencies of the parent and self reported scales. These issues require further investigation and it may be the case that certain questions would need to be altered or reworded in order to capture the intended constructs. The normative scores, cut-offs and bandings only differ slightly from those reported in other cultures. Convergent and discriminant validity and inter-rater agreement appear good however there are issues relating to stability as measured by test retest reliability. These findings clearly need to be replicated in other Chinese samples, including those from rural rather than urban settings. However until such data is available these results should be taken into account by clinicians and researchers using this instrument.

## Abbreviations

ADHD: Attention Deficit/Hyperactivity Disorder; PSQ: Conner's Parent Symptom Questionnaire; SDQ: Strengths and Difficulties Questionnaire

## Competing interests

YD has received research funding from Xi'an-Janssen Pharmaceutical Ltd, Eli Lilly and Company.

JK has no competing interests to declare.

DC is an advisory board member for Cephalon, Eli Lilly, Janssen Cilag, Shire and UCB and has received research funding from Eli Lilly and Janssen Cilag.

## Authors' contributions

YD and JK designed the study and collected the data, DC designed and conducted the analysis DC and YD wrote the paper. All authors revised and agreed the final paper.
